# Supplementary material for: Loss of PARP7 Increases Type I Interferon Signaling in EO771 Breast Cancer Cells and Prevents Mammary Tumor Growth by Increasing Antitumor Immunity
Source: Cancers (Basel). 2023 Jul 20;15(14):3689. doi: 10.3390/cancers15143689 (PMC10377955; doi:10.3390/cancers15143689)
Supplement: Supplementary file 1 [file cancers-15-03689-s001.zip › Revised_Original images for western blots.pdf]

Original images for western blots

Figure 1A: EO771 cells express PARP7

Lanes:
1) EO771 DMSO
2) EO771 100 nM RBN-2397, 24 h
3-4) Not relevant

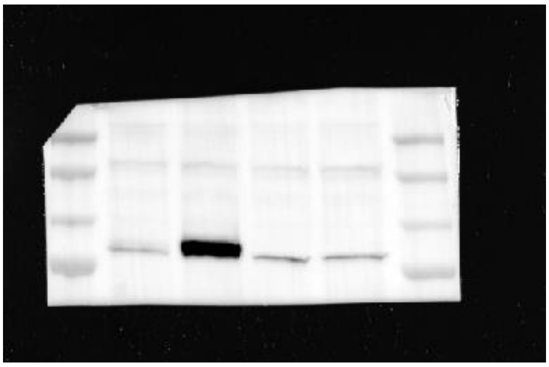

Panel 1: anti-PARP7

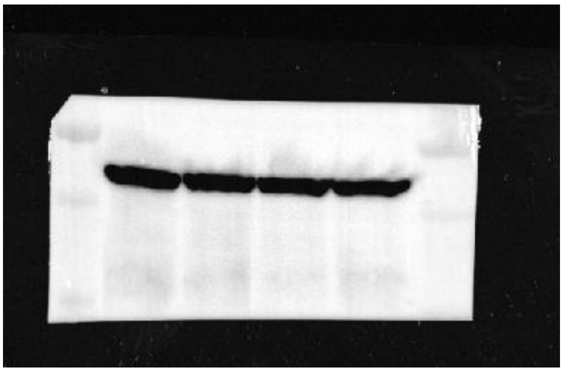

Panel 2: anti-β-actin

Figure 1B: NCI H1373 cells express PARP7

Lanes:
1) NCI H1373 DMSO
2) NCI H1373 100 nM RBN-2397, 24 h
3) NCI H1373 1000 nM RBN-2397, 24 h

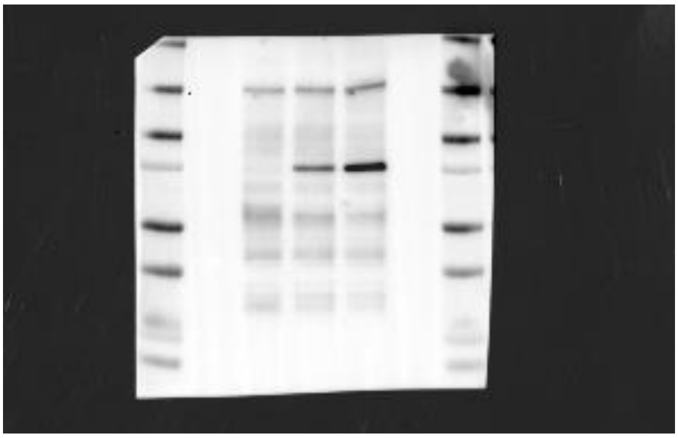

Panel 1: anti-PARP7

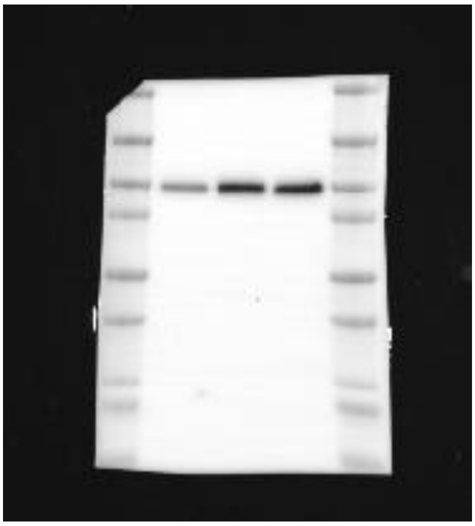

Panel 2: anti-AHR

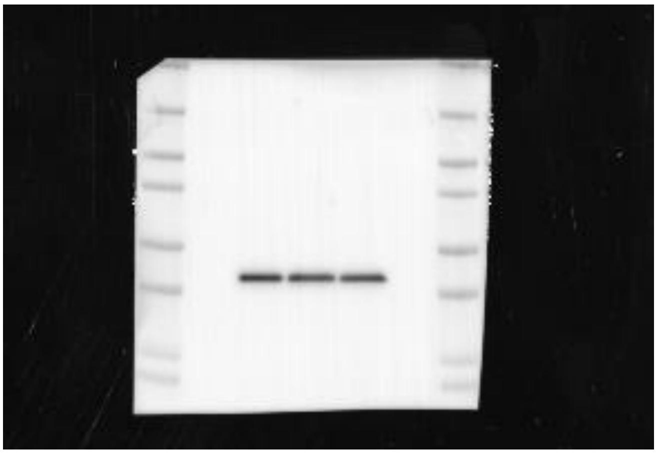

Panel 3: anti-β-actin

Figure 1C: PyMT, but not EO771 cells, express AHR

Lanes:
1) PyMT WT
2) PyMT AHR^{KO}
3) EO771 WT
4-8) Not relevant

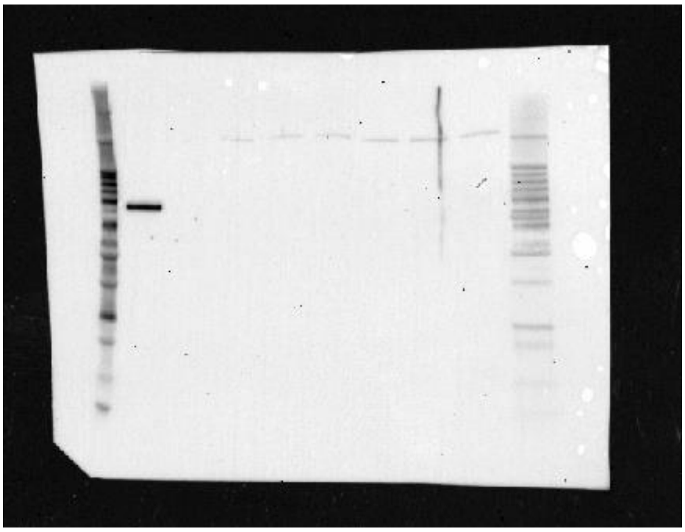

Panel 1: anti-AHR

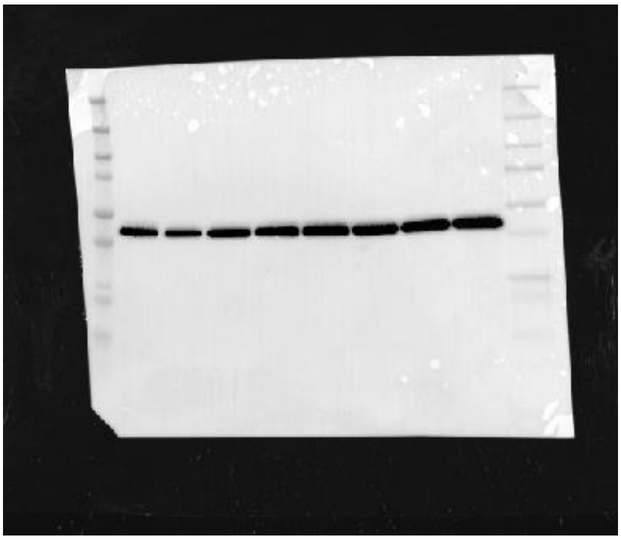

Panel 2: anti-β-actin

Figure 2A: EO771 Parp7^{KO} clones 1 and 2 do not express PARP7

Lanes:
1) EO771 WT DMSO
2) EO771 WT 100 nM RBN-2397, 24 h
3) EO771 Parp7^{KO} Clone 1 DMSO
4) EO771 Parp7^{KO} Clone 1 100 nM RBN-2397, 24 h
5) EO771 Parp7^{KO} Clone 2 DMSO
6) EO771 Parp7^{KO} Clone 2 100 nM RBN-2397, 24 h
7-12) Not relevant

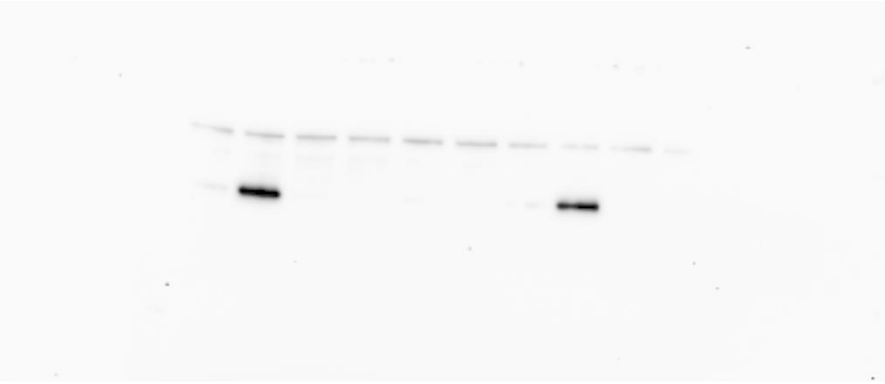

Panel 1: anti-PARP7

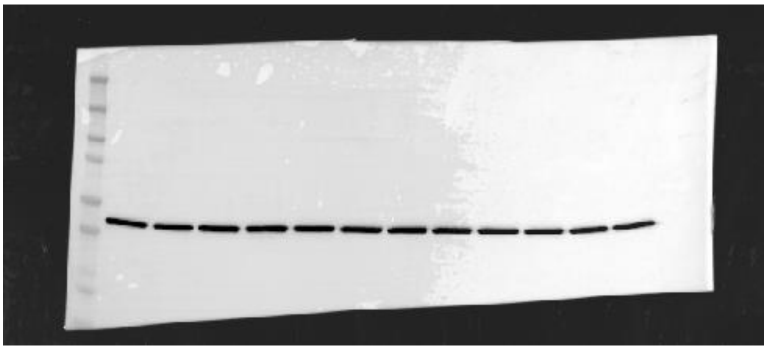

Panel 2: anti-β-actin

Figure 3A: Entire cGAS-STING-STAT1 pathway

Lanes:
1) EO771 WT DMSO
2) EO771 WT 10 µg/mL DMXAA, 2 h
3) EO771 WT 10 µg/mL DMXAA, 6 h
4) EO771 WT 10 µg/mL DMXAA, 24 h
5) EO771 WT 100 nM RBN-2397, 2 h
6) EO771 WT 100 nM RBN-2397, 6 h
7) EO771 WT 100 nM RBN-2397, 24 h
8) EO771 WT 10 µg/mL DMXAA + 100 nM RBN-2397, 2 h
9) EO771 WT 10 µg/mL DMXAA + 100 nM RBN-2397, 6 h
10) EO771 WT 10 µg/mL DMXAA + 100 nM RBN-2397, 24 h
11) EO771 Parp7^{KO} DMSO
12) EO771 Parp7^{KO} 10 µg/mL DMXAA, 2 h
13) EO771 Parp7^{KO} 10 µg/mL DMXAA, 6 h
14) EO771 Parp7^{KO} 10 µg/mL DMXAA, 24 h

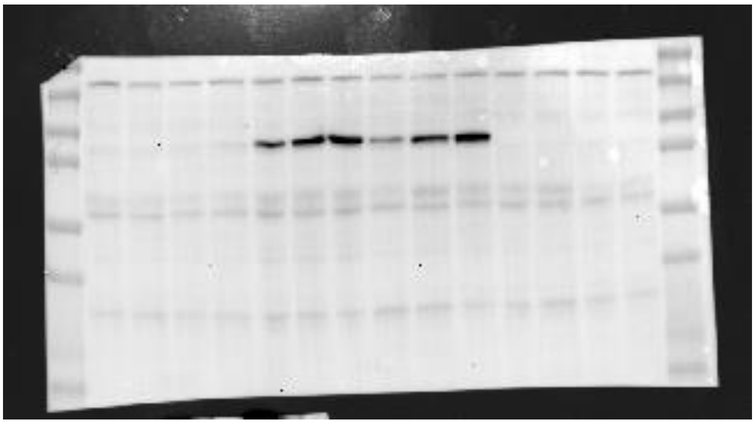

Panel 1: anti-PARP7

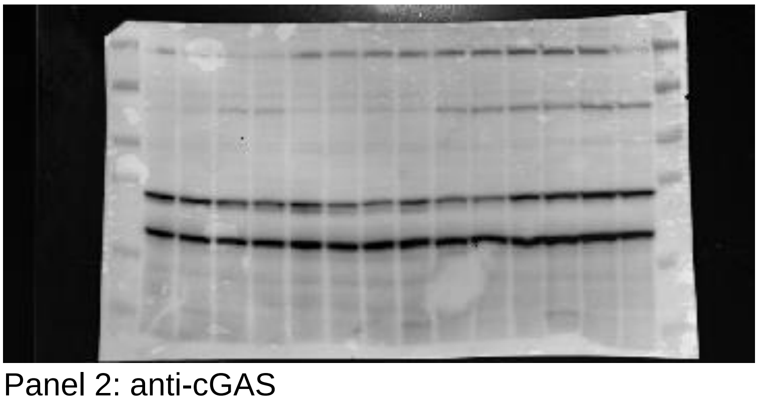

Panel 2: anti-cGAS

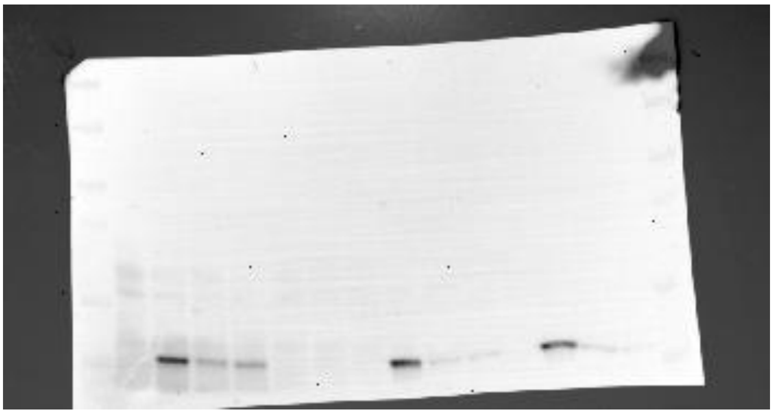

Panel 3: anti-pSTING

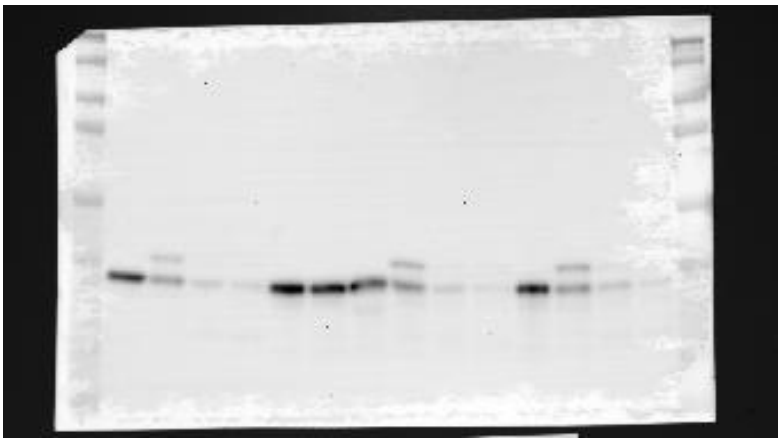

Panel 4: anti-STING

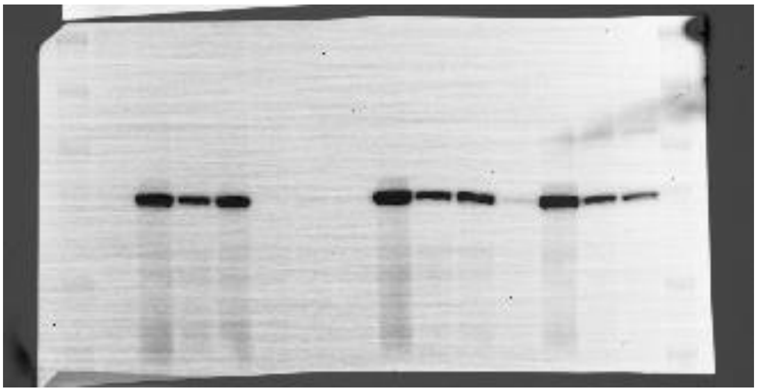

Panel 5: anti-pTBK1

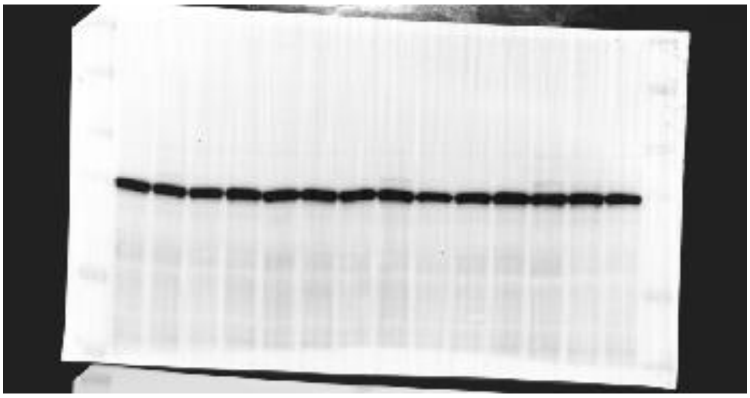

Panel 6: anti-TBK1

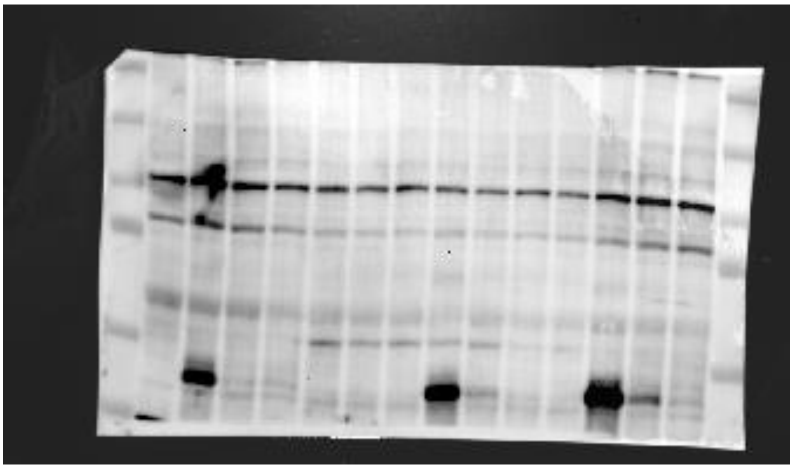
Panel 7: anti-pIRF3

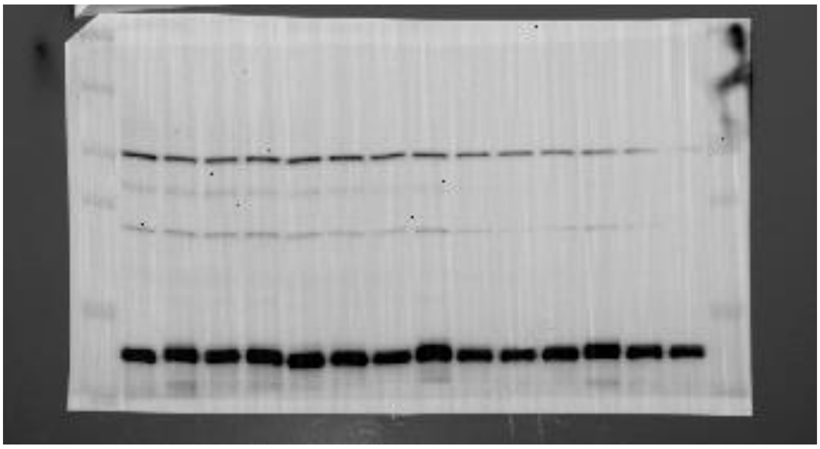
Panel 8: anti-IRF3

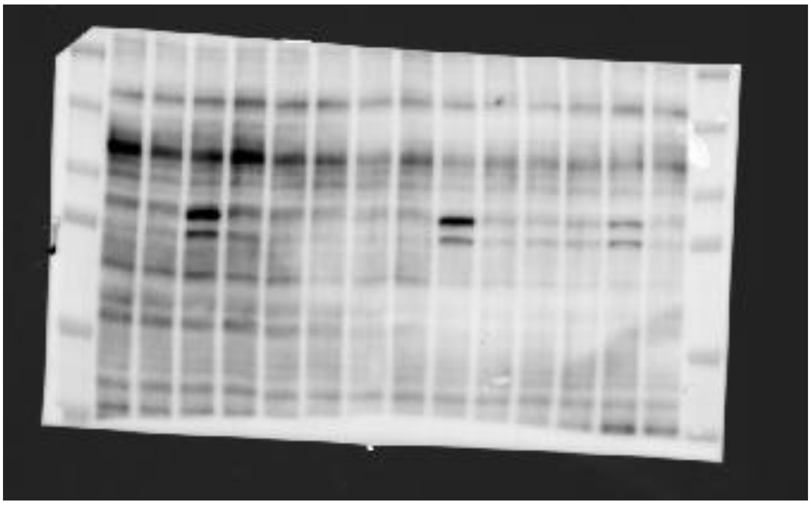
Panel 9: anti-pSTAT1

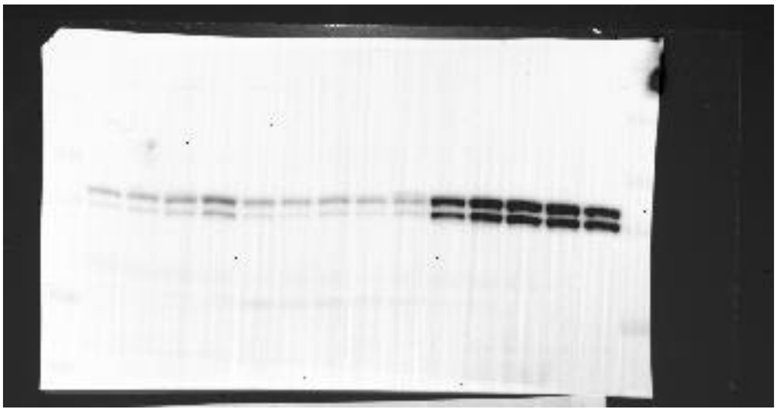
Panel 10: anti-STAT1

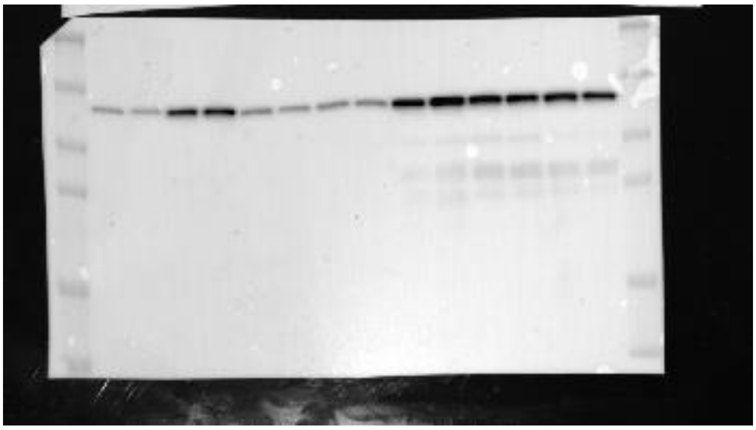
Panel 11: anti-STAT2

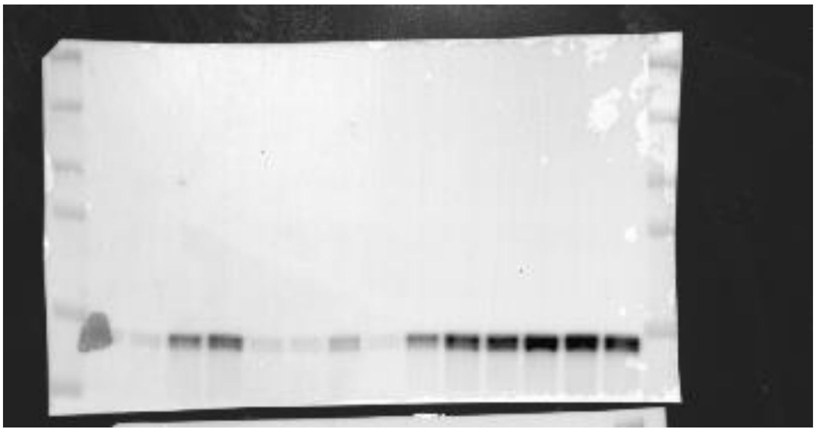
Panel 12: anti-IRF9

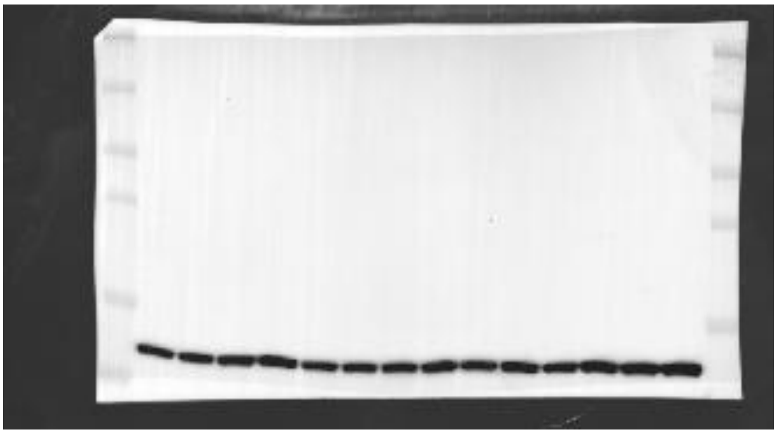

Panel 13: anti-β-actin

Figure 3D: Cytoplasmic and nuclear fractions showing ISGF3 subcellular localization after DMXAA treatment for 6 hours

Lanes:
1) EO771 WT Cytoplasm DMSO
2) EO771 WT Cytoplasm 10 µg/mL DMXAA, 6 h
3) EO771 WT Cytoplasm 100 nM RBN-2397, 6 h
4) EO771 WT Cytoplasm 10 µg/mL DMXAA + 100 nM RBN-2397, 6 h
5) EO771 Parp7^{KO} Cytoplasm DMSO
6) EO771 Parp7^{KO} Cytoplasm 10 µg/mL DMXAA, 6 h
7) EO771 WT Nucleus DMSO
8) EO771 WT Nucleus 10 µg/mL DMXAA, 6 h
9) EO771 WT Nucleus 100 nM RBN-2397, 6 h
10) EO771 WT Nucleus 10 µg/mL DMXAA + 100 nM RBN-2397, 6 h
11) EO771 Parp7^{KO} Nucleus DMSO
12) EO771 Parp7^{KO} Nucleus 10 µg/mL DMXAA, 6 h

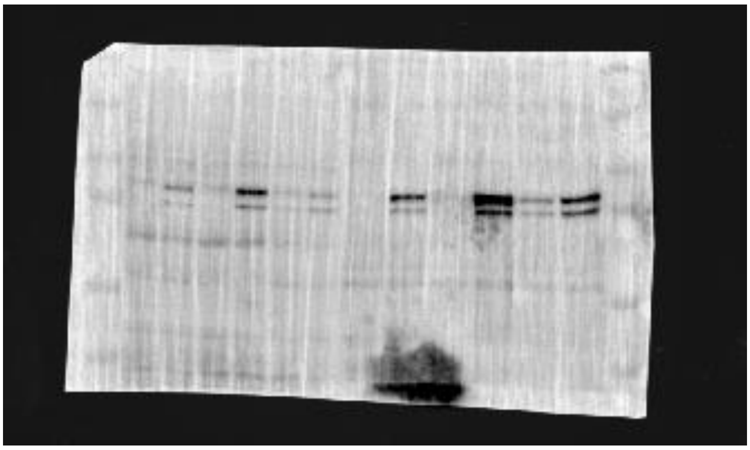

Panel 1: anti-pSTAT1

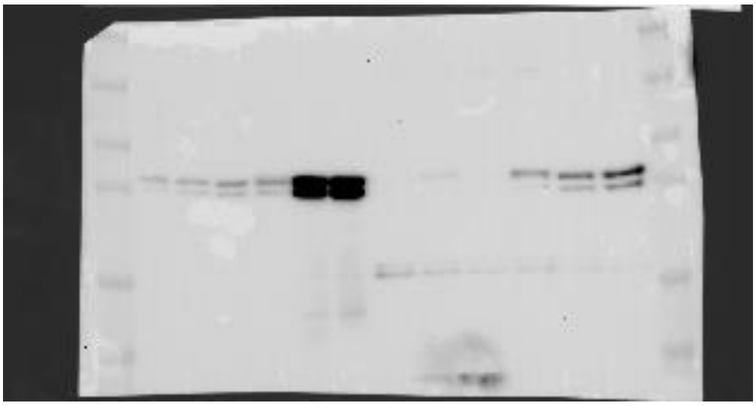

Panel 2: anti-STAT1

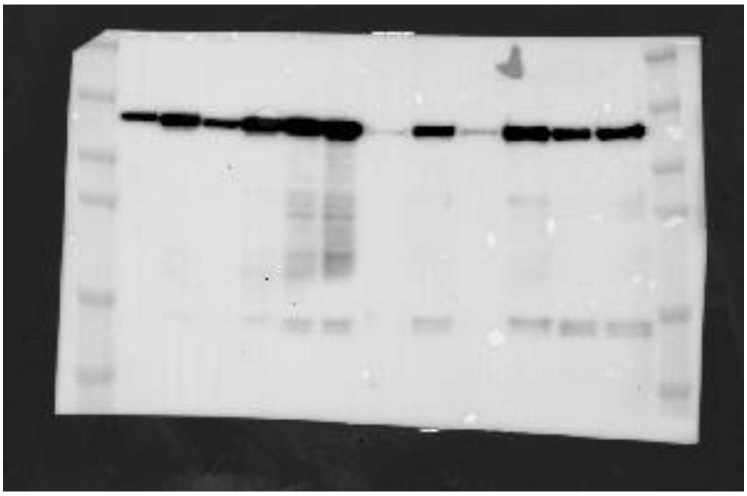
Panel 3: anti-STAT2

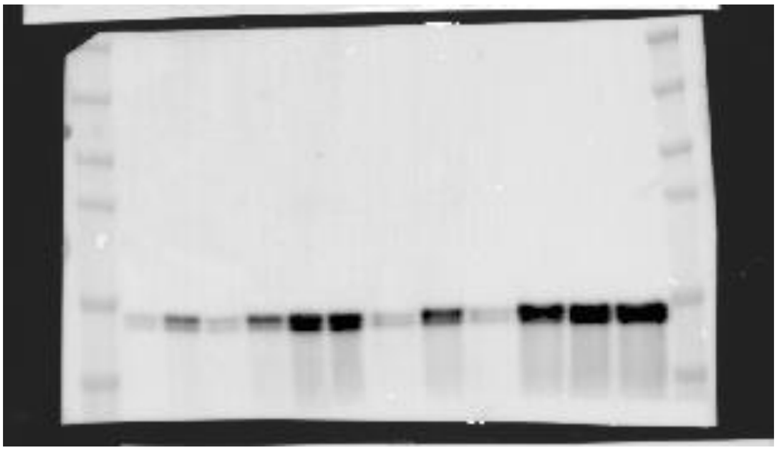
Panel 4: anti-IRF9

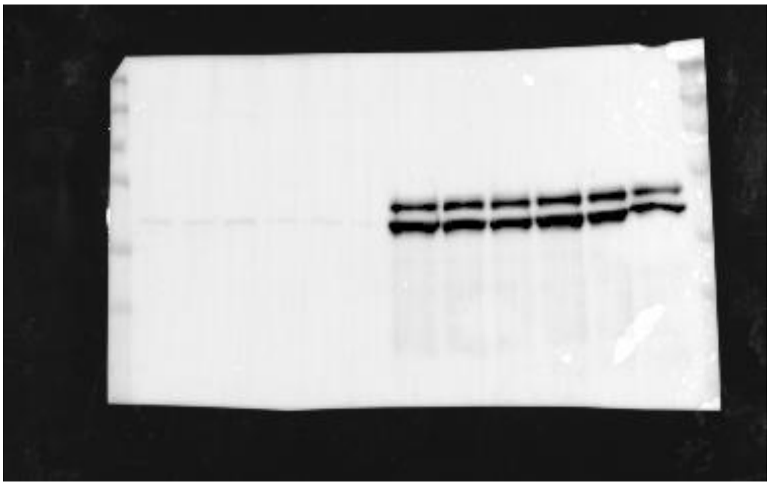
Panel 5: anti-Lamin A/C

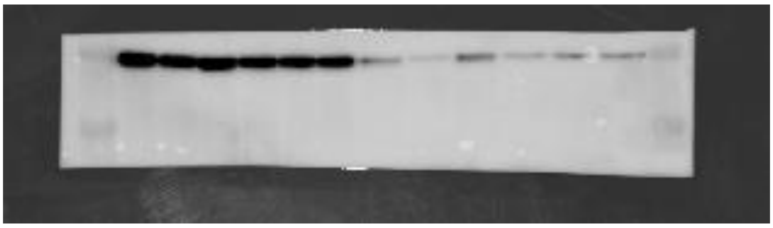

Panel 6: anti-Tubulin

Figure 4B: Endogenous levels of p50 and RelA

Lanes:
1) EO771 WT DMSO
2) EO771 WT 10 µg/mL DMXAA, 24 h
3) EO771 WT 100 nM RBN-2397, 24 h
4) EO771 WT 10 µg/mL DMXAA + 100 nM RBN-2397, 24 h
5) EO771 Parp7^{KO} DMSO
6) EO771 Parp7^{KO} 10 µg/mL DMXAA, 24 h

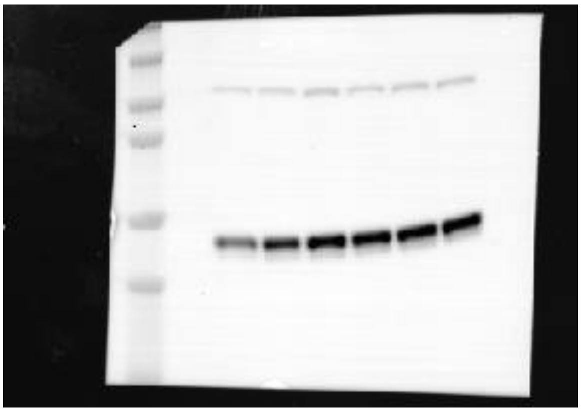

Panel 1: anti-p50

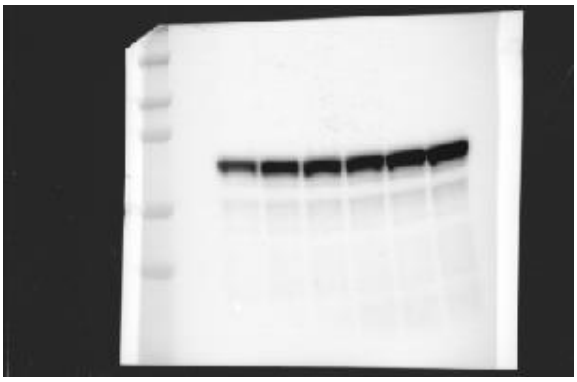

Panel 2: anti-RelA

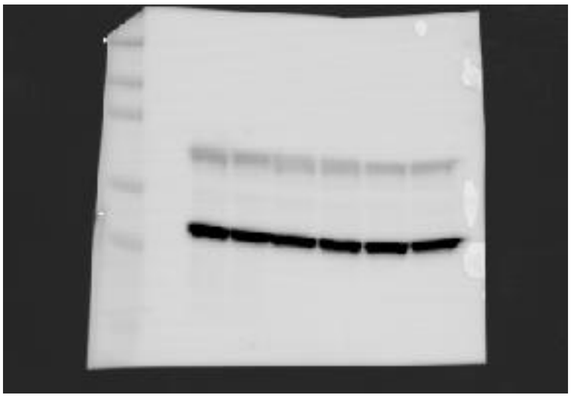

Panel 3: anti-β-actin

Figure 4E: Confirmation of TBK1 and RelA siRNA knockdown

Lanes:
1) EO771 WT Control
2) EO771 WT TBK1 siRNA
3) EO771 WT RelA siRNA
4) EO771 WT TBK + RelA siRNA
5) EO771 Parp7^{KO} Control
6) EO771 Parp7^{KO} TBK1 siRNA
7) EO771 Parp7^{KO} RelA siRNA
8) EO771 Parp7^{KO} TBK + RelA siRNA

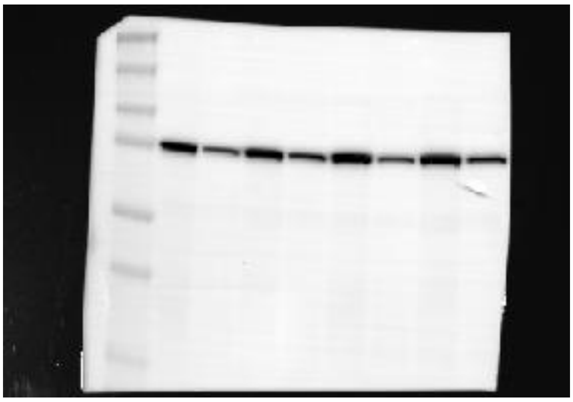

Panel 1: anti-TBK1

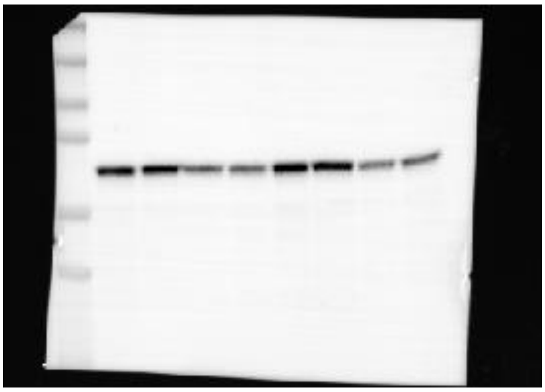

Panel 2: anti-RelA

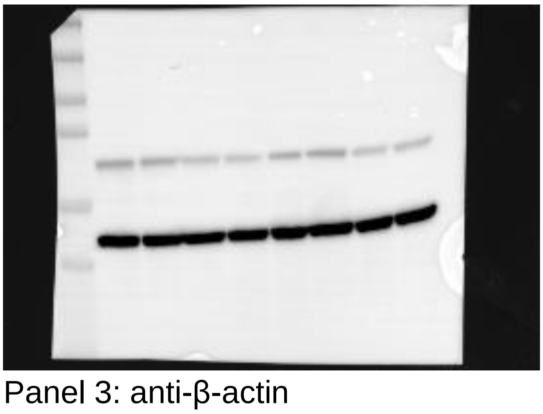

Panel 3: anti-β-actin

Figure 4G: Co-IP of overexpressed p50 and RelA

Lanes:
1) FLAG-p50 + GFP
2) FLAG-p50 + GFP-PARP7
3) FLAG-p50 + GFP-PARP7-H532A
4-8) Not relevant
9) FLAG-RelA + GFP
10) FLAG-RelA + GFP-PARP7
11) FLAG-RelA + GFP-PARP7-H532A

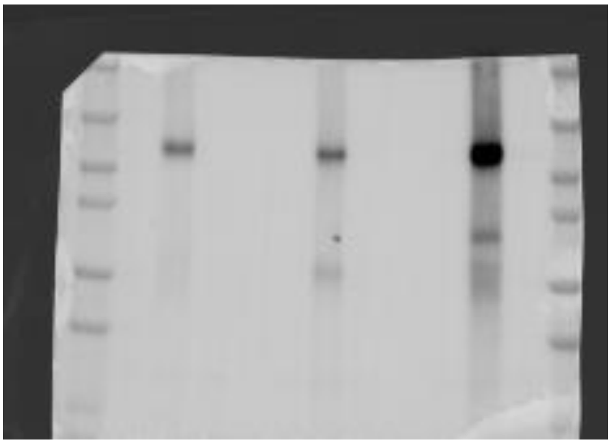
Panel 1: IP anti-ADPr

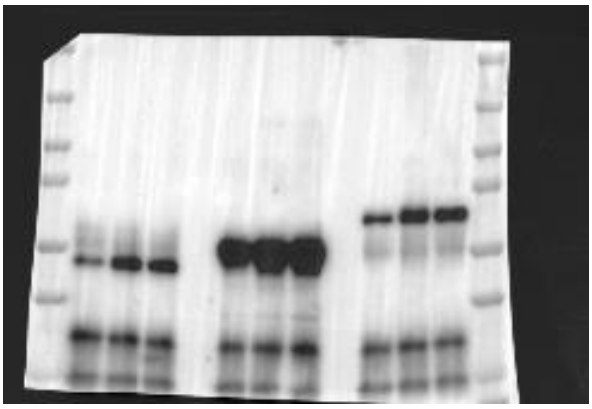
Panel 2: IP anti-FLAG

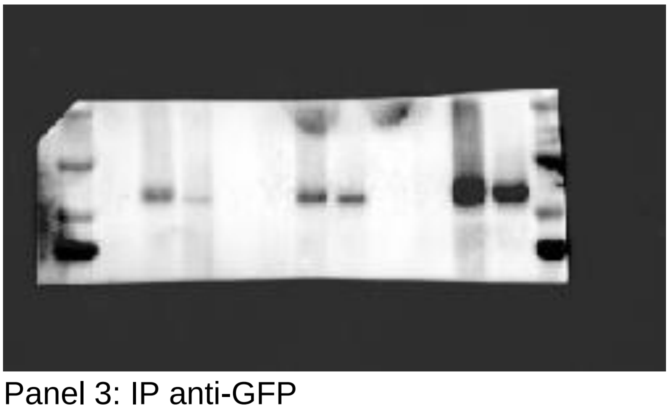
Panel 3: IP anti-GFP

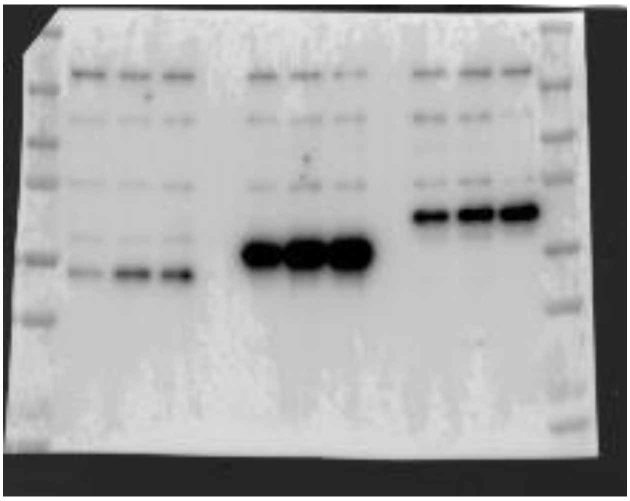
Panel 4: Input anti-FLAG

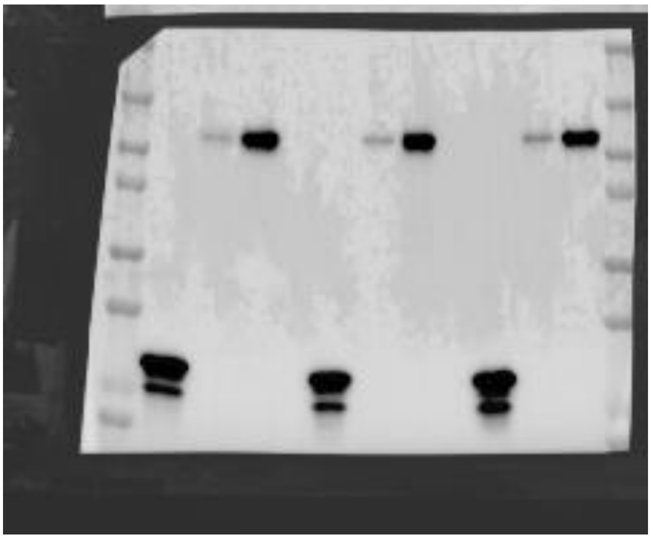
Panel 5: Input anti-GFP

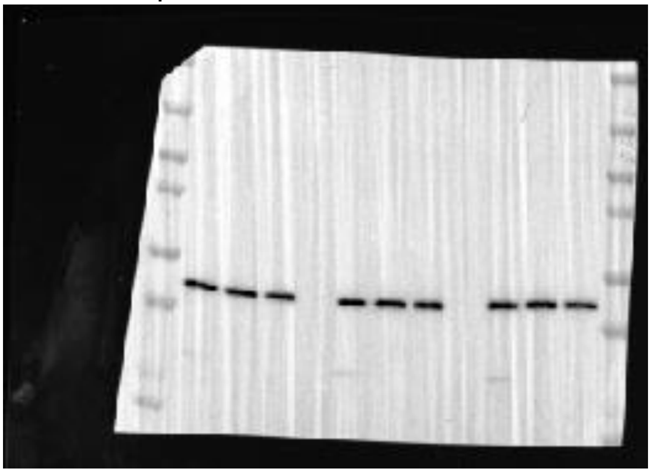
Panel 6: Input anti-β-actin

Supplementary Figure S5: Long term treatment with RBN-2397 in EO771 cells increases levels of STAT1, STAT2 and IRF9.

Lanes:
1) EO771 WT
2-4) Not relevant
5) EO771 WT + 100 nM RBN-2397, 24 h
6) EO771 WT + 100 nM RBN-2397, 48 h
7) EO771 Parp7^{KO}

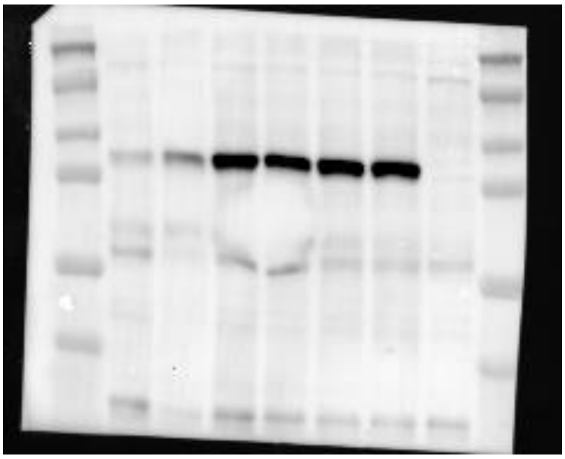

Panel 1: anti-PARP7

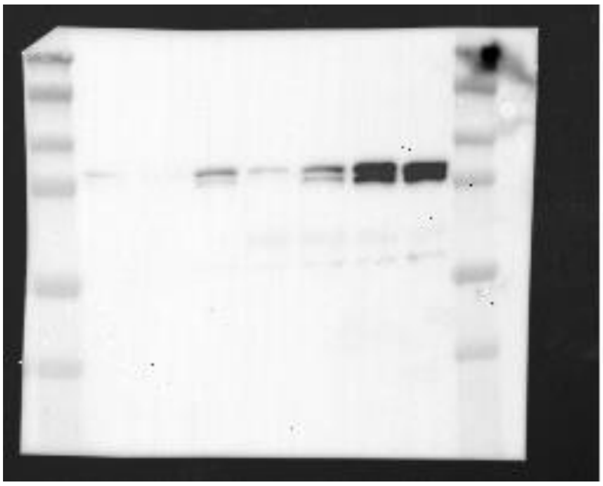

Panel 2: anti-STAT1

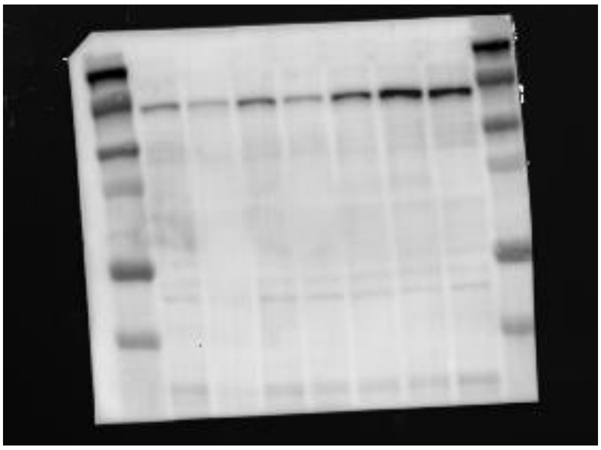
Panel 3: anti-STAT2

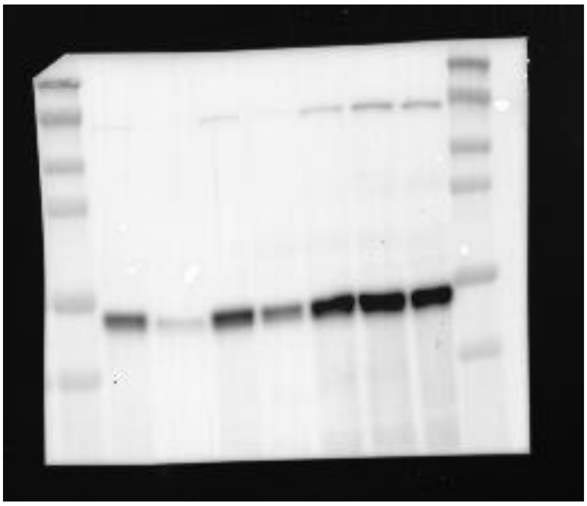
Panel 4: anti-IRF9

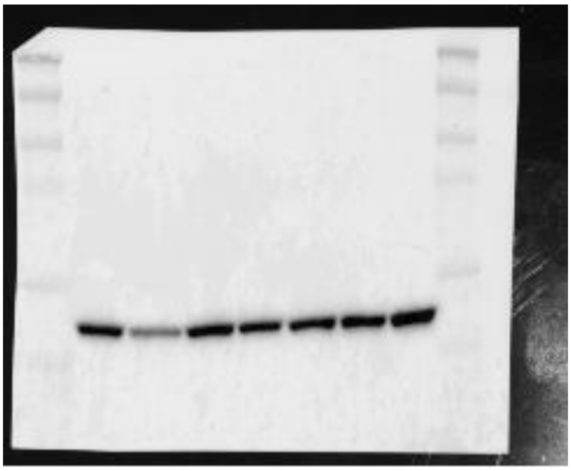
Panel 5: Panel 5: anti-β-actin

Supplementary Figure S6A: Upregulation of ISGF3 proteins in MEFs lacking functional PARP7

Lanes:
1) MEFs WT
2) MEFs WT 10 µg/mL DMXAA, 6 h
3) MEFs WT 10 µg/mL DMXAA, 24 h
4) MEFs WT 100 nM RBN-2397, 6 h
5) MEFs WT 100 nM RBN-2397, 24 h
6) MEFs WT 10 µg/mL DMXAA + 100 nM RBN-2397, 6 h
7) MEFs WT 10 µg/mL DMXAA + 100 nM RBN-2397, 24 h
8) MEFs Parp7^{H532A}
9) MEFs Parp7^{H532A} 10 µg/mL DMXAA, 6 h
10) MEFs Parp7^{H532A} 10 µg/mL DMXAA, 24 h
11-14) Not relevant

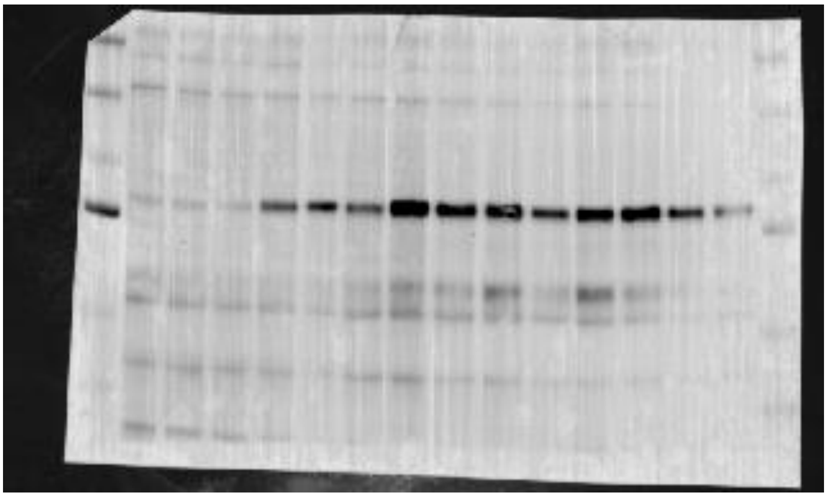

Panel 1: anti-PARP7

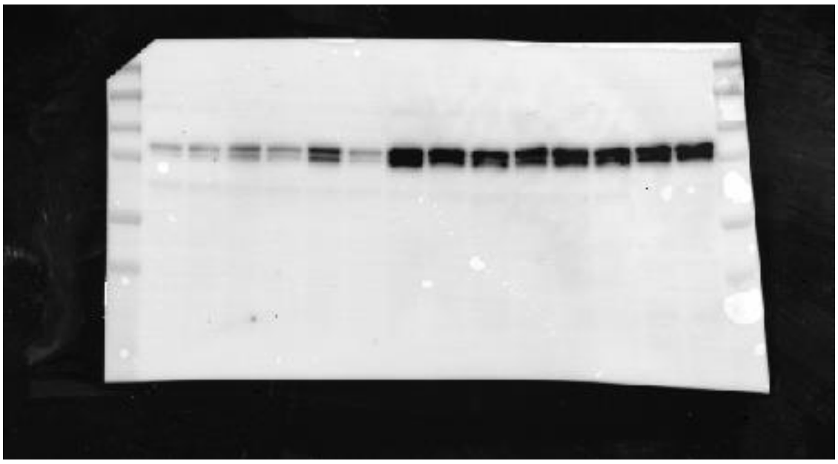

Panel 2: anti-STAT1

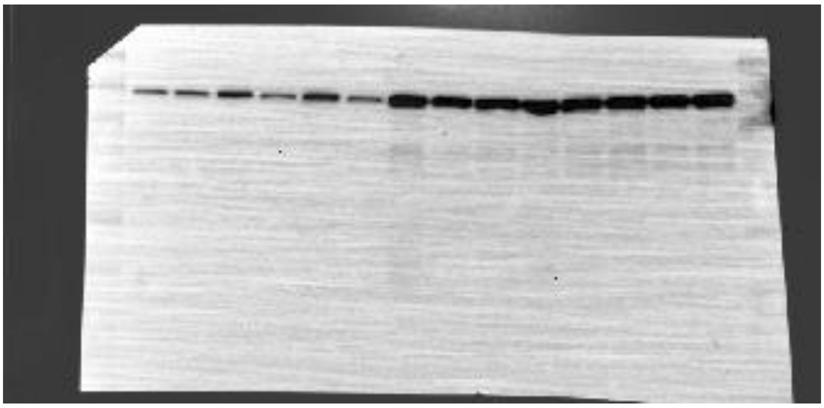
Panel 3: anti-STAT2

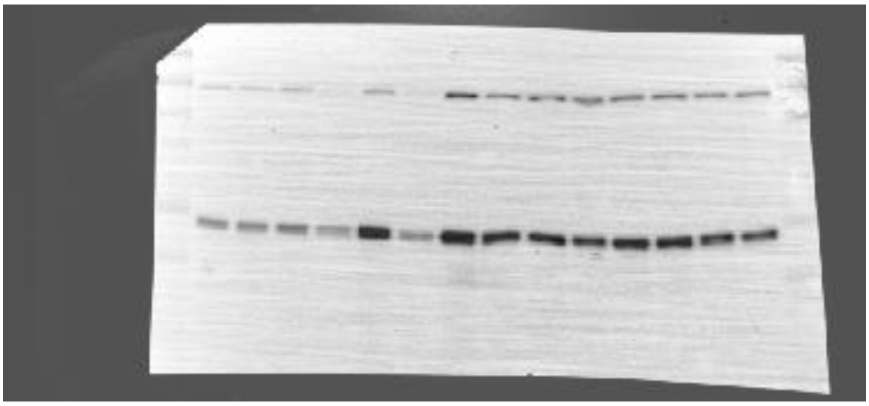
Panel 4: anti-IRF9

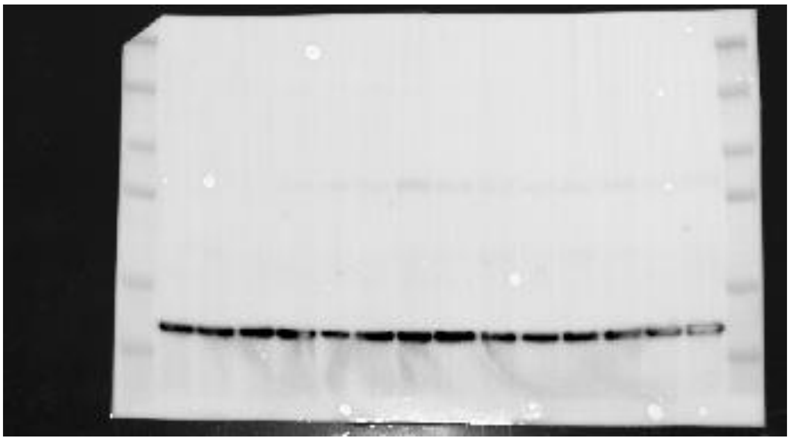
Panel 5: anti-β-actin
