# Supplementary material for: Loss of PARP7 Increases Type I Interferon Signaling in EO771 Breast Cancer Cells and Prevents Mammary Tumor Growth by Increasing Antitumor Immunity
Source: Cancers (Basel). 2023 Jul 20;15(14):3689. doi: 10.3390/cancers15143689 (PMC10377955; doi:10.3390/cancers15143689)
Supplement: Supplementary file 1 [file cancers-15-03689-s001.zip › Revised_Supplementary 2.pdf]

**Supplementary table S1.** Primers used for qPCR.

| | | |
|---|---|---|
| *Tbp* | Forward | GCACAGGAGCCAAGAGTGAA |
| | Reverse | TAGCTGGGAAGCCCAACTTC |
| *Ifnb1* | Forward | TGGGAGATGTCCTCAACTGC |
| | Reverse | CCAGGAGTAGCTGTTGTACT |
| *Cxcl10* | Forward | CCAAGTGCTGCCGTCATTTTC |
| | Reverse | GGCTCGCAGGGATGATTTCAA |
| *Pdl1* | Forward | CAGCAACTTCAGGGGGAGAG |
| | Reverse | TTTGCGGTATGGGGCATTGA |
| *Parp1* | Forward | GGCAGCCTGATGTTGAGGT |
| | Reverse | GCGTACTCCGCTAAAAAGTCAC |
| *Parp2* | Forward | TGGAAGGCGAGTGCTAAATG |
| | Reverse | GGGCTTTGCCCTTTAACAGC |
| *Parp3* | Forward | TGCGGCATGTTTGGAAAGTG |
| | Reverse | GTGCATGGTGGTAACATAGCC |
| *Parp4* | Forward | AGTGCTACAGCCCGTTTCC |
| | Reverse | CACAGCTTTCAGTTGTGGGC |
| *Tnks1* | Forward | CCCTGAGGCCTTACCTACCT |
| | Reverse | TCAAGACCCGCAACTTCTCC |
| *Tnks2* | Forward | TGATGGCAGAAAGTCAACTCCA |
| | Reverse | GCCACAGGTCCATTGCATTC |
| *Parp6* | Forward | GTACCTTGATGGACCAGAGCC |
| | Reverse | GCCAGCTCGGAACTTCTTGA |
| *Parp7* | Forward | AAAACCCCTGGAAATCAACC |
| | Reverse | GAATCTGCCACTGTCCCACT |
| *Parp8* | Forward | CACTTCCGAAACCACTTCGC |
| | Reverse | TAGGATACACTTTTGGGGCCG |
| *Parp9* | Forward | GCATTTGCTAAAGAGCACAAGGA |
| | Reverse | AAAGCACCACTATTACCGCTGA |
| *Parp10* | Forward | CGAAACGGCACACTCTACGG |
| | Reverse | GAGACCCTCAAAGGAGGTGC |
| *Parp11* | Forward | CAAACCCTTGTGGCTCCATTTCC |
| | Reverse | AGGCACTGATGGAGAAAGGAGC |
| *Parp12* | Forward | AAGTTCTGACCTGGTGAGCAGG |
| | Reverse | TGGTGACACAGGACCTGAACTG |
| *Parp13* | Forward | AGTAGTCCCACTGGTTTTGGC |
| | Reverse | TGCAACTCTGTGGCTTGTGG |
| *Parp14* | Forward | TGCTGAAGCTGTCAAGACTACA |
| | Reverse | ACAATGGCATGGGTCGTAGC |
| *Parp16* | Forward | CTTTGACCCGGCCAACTCC |
| | Reverse | AAACAGAGAAGTCTTGTTCAGGTG |
| *Stat1* | Forward | GCCTCTCATTGTCACCGAAGAAC |
| | Reverse | TGGCTGACGTTGGAGATCACCA |
| *Stat2* | Forward | GAACCAACTCTCCATTGCCTGG |
| | Reverse | CGTAAGAGGAGAACTGCCAGCT |
| *Irf9* | Forward | CAACATAGGCGGTGGTGGCAAT |
| | Reverse | GTTGATGCTCCAGGAACACTGG |
| *Myd88* | Forward | ACCTGTGTCTGGTCCATTGCCA |
| | Reverse | GCTGAGTGCAAACTTGGTCTGG |
| *Adar* | Forward | GCCAAAGACAGTGGTCAACCAG |
| | Reverse | GAACAAGGATGTTGCTGAGGAGC |

| | | |
|---|---|---|
| *Irf1* | Forward | TCCAAGTCCAGCCGAGACACTA |
| | Reverse | ACTGCTGTGGTCATCAGGTAGG |
| *Isg15* | Forward | CATCCTGGTGAGGAACGAAAGG |
| | Reverse | CTCAGCCAGAACTGGTCTTCGT |
| *Mx1* | Forward | TGGACATTGCTACCACAGAGGC |
| | Reverse | TTGCCTTCAGCACCTCTGTCCA |
| *Usp18* | Forward | GGAACCTGACTAAGGACCAGATC |
| | Reverse | GAGAGTGTGAGCAGTTTGCTCC |
| *Rela* | Forward | TCCTGTTCGAGTCTCCATGCAG |
| | Reverse | GGTCTCATAGGTCCTTTTGCGC |
| *Cd8a* | Forward | ACTACCAAGCCAGTGCTGCGAA |
| | Reverse | ATCACAGGCGAAGTCCAATCCG |
| *Ifng* | Forward | AGCAAGGCGAAAAAGGATGC |
| | Reverse | TCATTGAATGCTTGGCGCTG |
| *Ifnl2* | Forward | TCCCAGTGGAAGCAAAGGATTG |
| | Reverse | TCAAGCACCTCTTCTCGATGG |
| *Il6* | Forward | CTCTGCAAGAGACTTCCATCCA |
| | Reverse | AGTCTCCTCTCCGGACTTGT |
| *Arg1* | Forward | CATTGGCTTGCGAGACGTAGAC |
| | Reverse | GCTGAAGGTCTCTTCCATCACC |

**WT**

GAATTCGGCTTTGCAGATTTTTGCATAGCTTTTGAATCTTCATTTCTCAGTTTAAAAAAAGAAAATTGACCTGTAAGAGCTAACTATAATGCAAGCAGTGATTGCA
GATAGTTAACCATAGACTAACGCAAAATGTTTTAATGAATGAATGGGTTCAGTTGTCAGTTTTTAAATGATCATCTTTCTTCCTTTCCTGGTAGGATTTGTAGATA
CTGAGGCACAGTTGGGAGTTAATCACATC**ATG**GAAGTGGAAACCACTGAACCTGAGCCAGACTGTGTAGTACAGCCTCCTTCTCCTTCTGATGACTTTTCATGCCA
**AATGAGAATTTCTGAGAAGA**TCTCTCCATTGAAAACGTGTTTTAAGAAAAAACAGGAACAAAAAAGATTGGGAACTGGAACCCTGAGATCCTTGAGGCCAATATTA
AATACTTTGCTAGAATCTGGCTCACTTGATGGAGTTTTTAGAGCTAGAGACCAAAACAGAGATGAGAGCAGCTTACATGAACATATAGTGAAAAAACCCCTGGAAA
TCAACCCATCGTGTCCACCAGCAGAAAACAGTATGCCTGTCCTGATTCCTGATGGGACAAATGTTGAGGGCCAATTACCAGAAGCGCATCCTTCTACAGATGCTCC
AGAACAGGGGGTTCCAATCCAAGACCACAGTTTTCCACCAGAAACCATCAGTGGGACAGTGGCAGATTCTACAACAGGACACTTCCAAACTGACCTTTTGCATCCT
GTTTCAGGTGATGTTCCTACAAGTCCTGACTGCGTAGATAAAGTTATGGATTATGTACCAGGAGCTTTCCAAGACAAAAGCCGAATTC

**Missing 320, Clone 1: 9, Clone 2: 7**

GAATTCGGCTTTGCAGATTTTTGCATAGCTTTTGAATCTTCATTTCTCAGTTTAAAAAAAGAAAATTGACCTGTAAGAGCTAACTATAATGCAAGCAGTGATTGCA
GATAGTTAACCATAGACTAACGCAAAATGTTTTAATGAATGAATGGGTTCAGTTGTCA*gtttttaaatgatcatctttcttcctttcctggtaggatttgtagata*
*ctgaggcacagttgggagttaatcacatcatggaagtggaaaccactgaacctgagccagactgtgtagtacagcctccttctccttctgatgacttttcatgcca*
***aatgagaatttctgagaaga****tctctccattgaaaacgtgttttaagaaaaaacaggaacaaaaaagattgggaactggaaccctgagatccttgaggccaatatta*
*aatactttgctagaatctggctcacttgatggagtttttagagctagagaccaaaacaga*GATGAGAGCAGCTTACATGAACATATAGTGAAAAAACCCCTGGAAA
TCAACCCATCGTGTCCACCAGCAGAAAACAGTATGCCTGTCCTGATTCCTGATGGGACAAATGTTGAGGGCCAATTACCAGAAGCGCATCCTTCTACAGATGCTCC
AGAACAGGGGGTTCCAATCCAAGACCACAGTTTTCCACCAGAAACCATCAGTGGGACAGTGGCAGATTCTACAACAGGACACTTCCAAACTGACCTTTTGCATCCT
GTTTCAGGTGATGTTCCTACAAGTCCTGACTGCGTAGATAAAGTTATGGATTATGTACCAGGAGCTTTCCAAGACAAAAGCCGAATTC

**Missing 274, Clone 1: 2, Clone 2: 1**

GAATTCGGCTTTGCAGATTTTTGCATAGCTTTTGAATCTTCATTTCTCAGTTTAAAAAAAGAAAATTGACCTGTAAGAGCTAACTATAATGCAAGCAGTGATTGCA
GATAGTTAACCATAGACTAACGCAAAATGTTTTAATGAATGAATGGGTTCAGTTGTCAGTTTTTAAATGATCATCTTTCTTCCTTTC*ctggtaggatttgtagata*
*ctgaggcacagttgggagttaatcacatcatggaagtggaaaccactgaacctgagccagactgtgtagtacagcctccttctccttctgatgacttttcatgcca*
***aatgagaatttctgagaaga****tctctccattgaaaacgtgttttaagaaaaaacaggaacaaaaaagattgggaactggaaccctgagatccttgaggccaatatta*
*aatactttgctagaatctggctcacttgatggagtttttagag*CTAGAGACCAAAACAGAGATGAGAGCAGCTTACATGAACATATAGTGAAAAAACCCCTGGAAA
TCAACCCATCGTGTCCACCAGCAGAAAACAGTATGCCTGTCCTGATTCCTGATGGGACAAATGTTGAGGGCCAATTACCAGAAGCGCATCCTTCTACAGATGCTCC
AGAACAGGGGGTTCCAATCCAAGACCACAGTTTTCCACCAGAAACCATCAGTGGGACAGTGGCAGATTCTACAACAGGACACTTCCAAACTGACCTTTTGCATCCT
GTTTCAGGTGATGTTCCTACAAGTCCTGACTGCGTAGATAAAGTTATGGATTATGTACCAGGAGCTTTCCAAGACAAAAGCCGAATTC

**Missing 352, Clone 1: 6, Clone 2: 16**

GAATTCGGCTTTGCAGATTTTTGCATAGCTTTTGAATCTTCATTTCTCAGTTTAAAAAAAGAAAATTGACCTGTAAGAGCTAACTATAATGCAAGCAGTGATTGCA
GATAGTTAACCAT*agactaacgcaaaatgttttaatgaatgaatgggttcagttgtcagtttttaaatgatcatctttcttcctttcctggtaggatttgtagata*
*ctgaggcacagttgggagttaatcacatcatggaagtggaaaccactgaacctgagccagactgtgtagtacagcctccttctccttctgatgacttttcatgcca*
***aatgagaatttctgagaaga****tctctccattgaaaacgtgttttaagaaaaaacaggaacaaaaaagattgggaactggaaccctgagatccttgaggccaatatta*
*aatactttgctagaatctggctcacttgatggagtttttagagctag*AGACCAAAACAGAGATGAGAGCAGCTTACATGAACATATAGTGAAAAAACCCCTGGAAA
TCAACCCATCGTGTCCACCAGCAGAAAACAGTATGCCTGTCCTGATTCCTGATGGGACAAATGTTGAGGGCCAATTACCAGAAGCGCATCCTTCTACAGATGCTCC
AGAACAGGGGGTTCCAATCCAAGACCACAGTTTTCCACCAGAAACCATCAGTGGGACAGTGGCAGATTCTACAACAGGACACTTCCAAACTGACCTTTTGCATCCT
GTTTCAGGTGATGTTCCTACAAGTCCTGACTGCGTAGATAAAGTTATGGATTATGTACCAGGAGCTTTCCAAGACAAAAGCCGAATTC

**Missing 1, Clone 1: 2, Clone 2: 2**

GAATTCGGCTTTGCAGATTTTTGCATAGCTTTTGAATCTTCATTTCTCAGTTTAAAAAAAGAAAATTGACCTGTAAGAGCTAACTATAATGCAAGCAGTGATTGCA
GATAGTTAACCATAGACTAACGCAAAATGTTTTAATGAATGAATGGGTTCAGTTGTCAGTTTTTAAATGATCATCTTTCTTCCTTTCCTGGTAGGATTTGTAGATA
CTGAGGCACAGTTGGGAGTTAATCACATC**ATG**GAAGTGGAAACCACTGAACCTGAGCCAGACTGTGTAGTACAGCCTCCTTCTCCTTCTGATGACTTTTCATGCCA
**AAT-AGAATTTCTGAGAAGA**TCTCTCCATTGAAAACGTGTTTTAAGAAAAAACAGGAACAAAAAAGATTGGGAACTGGAACCCTGAGATCCTTGAGGCCAATATTA
AATACTTTGCTAGAATCTGGCTCACTTGATGGAGTTTTTAGAGCTAGAGACCAAAACAGAGATGAGAGCAGCTTACATGAACATATAGTGAAAAAACCCCTGGAAA
TCAACCCATCGTGTCCACCAGCAGAAAACAGTATGCCTGTCCTGATTCCTGATGGGACAAATGTTGAGGGCCAATTACCAGAAGCGCATCCTTCTACAGATGCTCC
AGAACAGGGGGTTCCAATCCAAGACCACAGTTTTCCACCAGAAACCATCAGTGGGACAGTGGCAGATTCTACAACAGGACACTTCCAAACTGACCTTTTGCATCCT
GTTTCAGGTGATGTTCCTACAAGTCCTGACTGCGTAGATAAAGTTATGGATTATGTACCAGGAGCTTTCCAAGACAAAAGCCGAATTC

**Supplementary figure S1.** Indels resulting in reading frame errors in the *Parp7* gene. Genomic DNA was isolated from multiple selected and expanded clones, and the region surrounding the gRNA target site of *Parp7* (labeled in red) was amplified and sequenced. The sequences above show the deletions (and frequencies) from clone 1 and 2 which were used in the study.

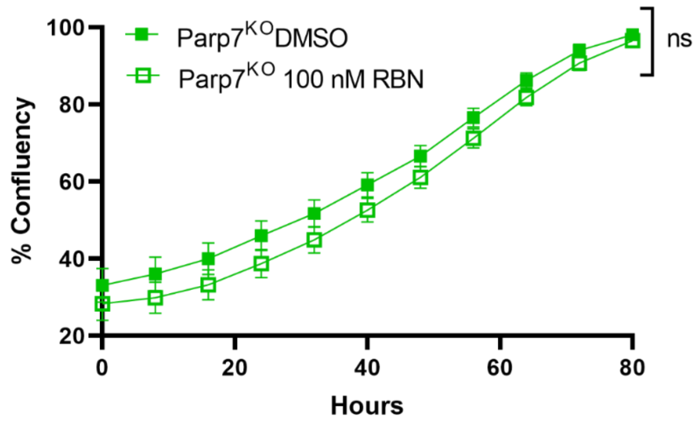


**Supplementary figure S2.** Treatment with RBN-2397 does not significantly affect proliferation in EO771 Parp7$^{KO}$ cells. Cells were dosed with 100 nM of RBN-2397, and proliferation was measured as an increase in confluency over time. *n*=4.

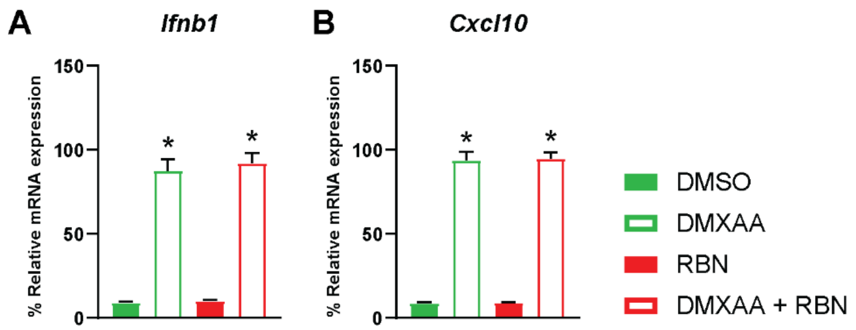


**Supplementary figure S3.** Treatment with RBN-2397 does not affect type I IFN signaling in EO771 Parp7$^{KO}$ cells. **(A-B)** Expression levels of *Ifnb1* and *Cxcl10* are significantly higher in response to DMXAA, but RBN-2397 does not further elevate this. Cells were treated with 10 µg/mL of DMXAA and/or 100 nM of RBN-2397 for 2 h, and expression levels were determined by RT-qPCR. * denotes statistical significance from the DMSO treated samples. *n*=3.

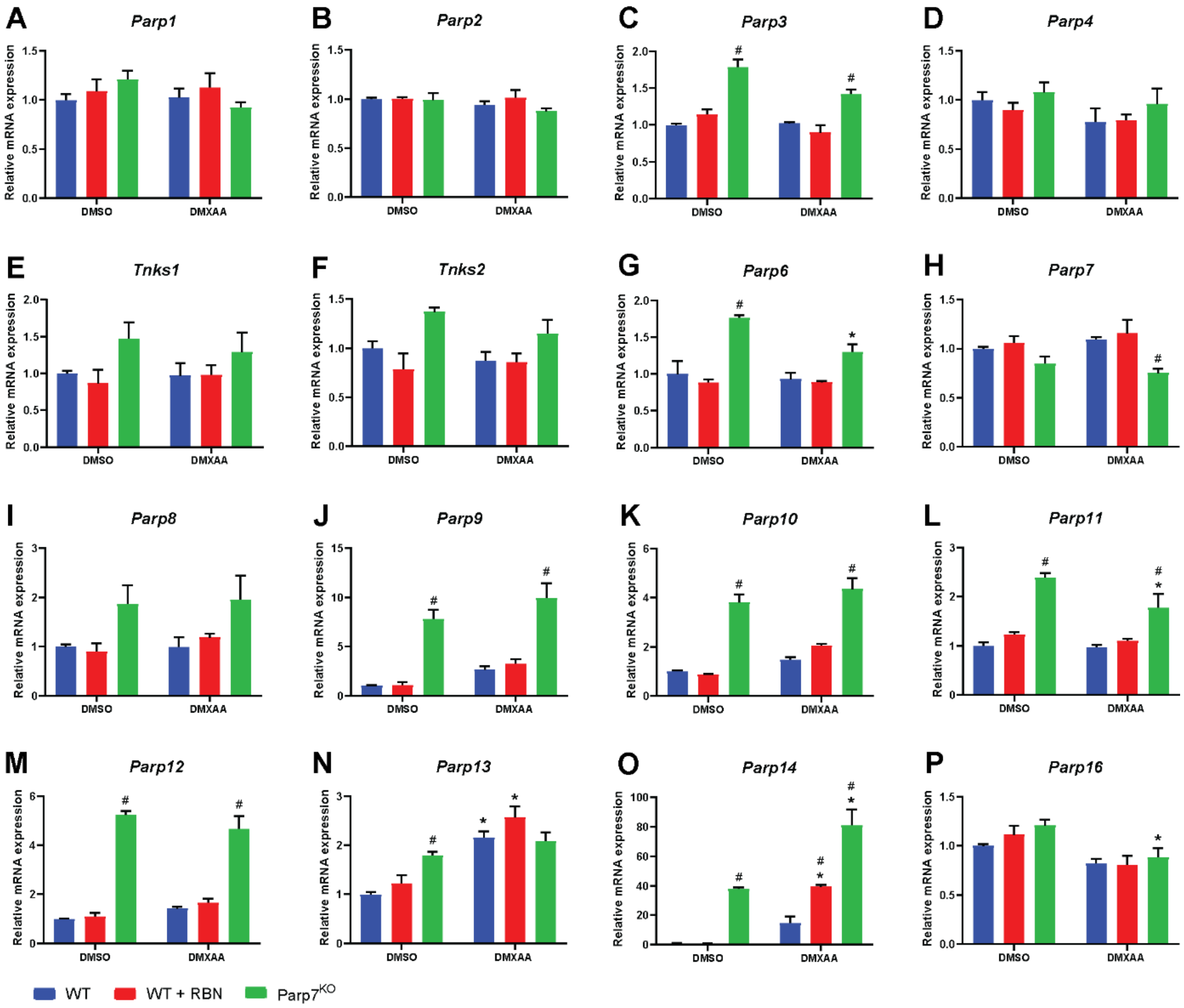


**Supplementary figure S4.** Expression levels of different PARPs in the ARTD family in response to DMXAA and/or RBN-2397 in EO771 WT and DMXAA in the Parp7$^{KO}$ cells. **(A-B, D-F, I)** Levels of *Parp1*, *Parp2*, *Parp4*, *Tnks1* (Parp5a), *Tnks2* (Parp5b) and *Parp8* levels are not significantly changed in response to treatment or knockout of PARP7. **(C, J-K, M)** *Parp3*, *Parp9*, *Parp10* and *Parp12* levels are significantly upregulated in Parp7$^{KO}$ cells indepedent of treatment. **(G)** *Parp6* levels are significantly upregulated in untreated Parp7$^{KO}$ cells, but lower after treatment with DMXAA. **(H)** Levels of *Parp7* are significantly lower in DMXAA treated Parp7$^{KO}$ cells. **(L)** *Parp11* levels are significantly higher in Parp7$^{KO}$ cells, but lower after treatment with DMXAA compared to DMSO. **(N)** *Parp13* levels are significantly higher in DMSO treated Parp7$^{KO}$ cells and in WT cells treated with DMXAA and DMXAA + RBN-2397. **(O)** *Parp14* levels are significantly higher in Parp7$^{KO}$ cells and increase in response to DMXAA. In the WT cells, co-treatment with DMXAA and RBN-2397 further increases *Parp14* levels. **(P)** *Parp16* levels are significantly decreased in DMXAA-treated Parp7$^{KO}$ cells. Cells were treated with 10 µg/mL of DMXAA and/or 100 nM of RBN-2397 for 2 h, and expression levels were determined by RT-qPCR. * denotes statistical significance compared to the DMSO-treated samples, while # denotes significance between the genotypes of the cells. The data are the summary of two independent experiments.

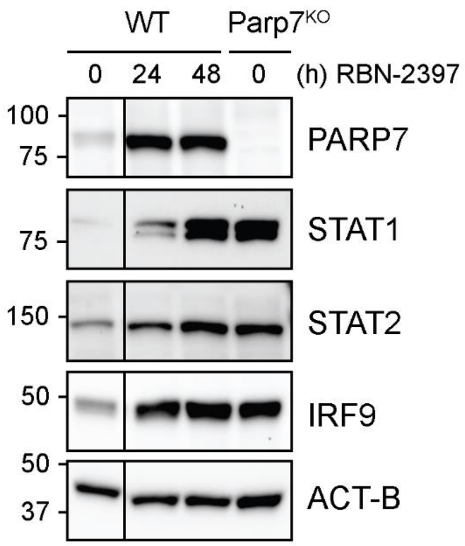


**Supplementary figure S5.** Long term treatment with RBN-2397 increases protein levels of STAT1, STAT2 and IRF9. Cells were treated with 100 nM of RBN-2397 for 24 and 48 h, and proteins were visualized together with negative controls. Data are representative of 2 independent replicates.

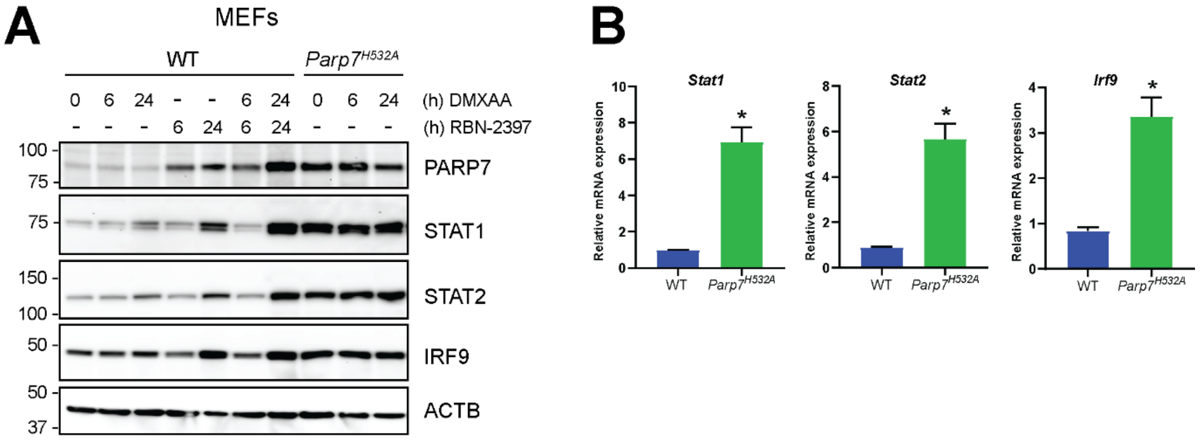


**Supplementary figure S6.** MEFs isolated from *Parp7*$^{H532A}$ mice display increased expression levels of ISGF3. **(A)** Western blot showing increased protein levels of STAT1, STAT2 and IRF9 in cells lacking functional PARP7. PARP7 protein levels are stabilized in *Parp7*$^{H532A}$ cells. Cells were treated with 10 µg/mL of DMXAA (+/- 100 nM RBN-2397) for 6 and 24 h. **(B)** Cells lacking functional PARP7 display increased mRNA levels of *Stat1*, *Stat2* and *Irf9*. * denotes statistical significance ($p < 0.05$). $n$=3.

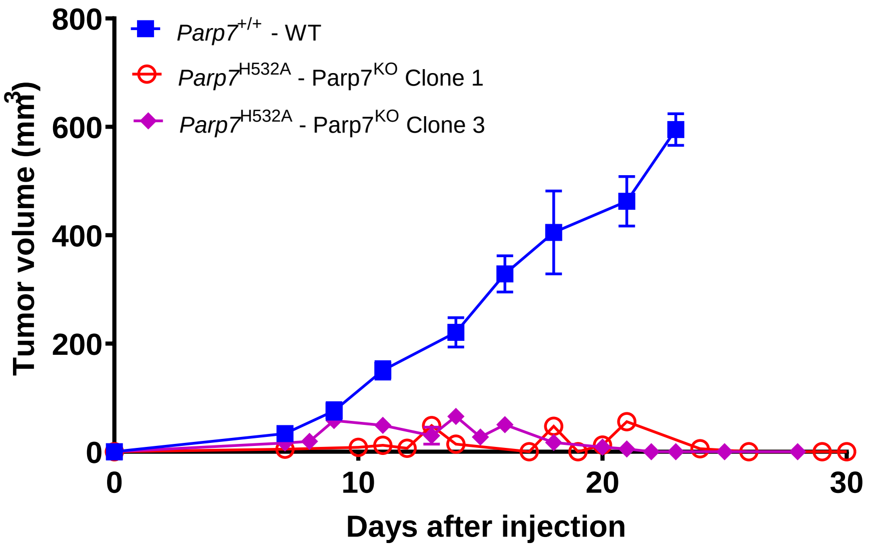


**Supplementary figure S7.** Two additional Parp7$^{KO}$ clones (1 and 3) also fail to develop tumors in mice lacking functional PARP7. *Parp7$^{H532A}$* mice were injected with either EO771 Parp7$^{KO}$ clone 1 (described in the study, Figure 2A-C) or 3, and tumors were measured three times a week until day 30. *n*=6 for clone 1, *n*=4 for clone 3.
